# Postoperative myelitis subsequent to cervical spine radiofrequency ablation: a case report

**DOI:** 10.3389/fsurg.2025.1570965

**Published:** 2025-06-24

**Authors:** Cuiyu Xu, Maoyu Ding, Yumei Yang, Chunke Dong, Xiaolong Xu, Zhicheng Qu, Wei Guo, Ying Cao

**Affiliations:** ^1^ Beijing Hospital of Traditional Chinese Medicine, Capital Medical University, Beijing, China; ^2^ Emergency and Critical Care Centre, Beijing Hospital of Traditional Chinese Medicine, Capital Medical University, Beijing, China; ^3^ Department of Orthopedics, Beijing Hospital of Traditional Chinese Medicine, Capital Medical University, Beijing, China; ^4^ Beijing Institute of Chinese Medicine, Beijing, China

**Keywords:** cervical spine radiofrequency ablation, myelitis, complications, case report, radiofrequency ablation

## Abstract

Commonly used minimally invasive treatment for cervical spondylotic diseases, cervical spine radiofrequency ablation (RFA) provides benefits of faster recovery and less trauma. However, data on the monitoring and management of postoperative complications, especially severe adverse events like myelitis, is limited. This case report details a 75-year-old female patient with a medical history of hypertension, cerebral infarction, and hyperlipidemia who subsequently developed myelitis after undergoing cervical spine radiofrequency ablation (RFA). The patient received effective treatment following a sequence of diagnostic and therapeutic measures, highlighting the risk of severe complications and the vital necessity of maintaining rigorous aseptic protocols during the procedure. This report extensively discusses the etiology of myelitis in this patient and explores the direct or indirect role of RFA in the development of myelitis.

## Background

With neck pain and stiffness, cervical spondylosis is a common disorder affecting 21.3% of the population according to the China Health Statistics Yearbook 2019 (1, 2). One less invasive substitute for conventional surgical treatments is radiofrequency ablation (RFA) of the cervical spine. This procedure uses electrical currents to deliver heat through an electrode placed near a nerve, which the nociceptive pathway is thought to arise, to interrupt pain impulses (3, 4). Although the reported post–RFA complication rate is low, estimated at 0.8%, this may be due to underreporting and insufficient consideration of possible side effects. Thus, it is crucial to be vigilant about potential complications (5).

## Case presentation

A 75-year-old woman with a medical history of hypertension, cerebral infarction, and hyperlipidemia presented with a three-month history of neck pain (Visual Analogue Scale, VAS: 8/10) and dizziness exacerbated by neck rotation. The preoperative neurological evaluation indicated the absence of motor deficits (bilateral limb muscle strength: grade 5/5), preserved sensation, and symmetrical deep tendon reflexes. Significant physical findings comprised tenderness over the spinous processes from C4 to C7, along with bilateral positive Spurling test, right-sided positive Eaton test, and bilateral positive Fenz sign. The cervical MRI indicated spinal stenosis from C3 to C7 and bilateral neuroforaminal narrowing from C4 to C7. The patient was diagnosed with cervical spondylotic radiculopathy based on clinical symptoms, physical signs, and imaging findings.

The procedure was tailored to the patient's regional pain distribution and anatomical pathology. The right C5 spinal nerve root and right C5/6 facet joint were targeted in order to address her right-sided suprascapular pain. This approach was based on the dermatomal distribution of the C5 nerve roots and imaging evidence. Concurrently, the bilateral C2 spinal nerves (posterior branches) and C2/3 facet joints were ablated to alleviate bilateral occipital and retroauricular pain, consistent with the clinical diagnosis of greater occipital neuralgia and facet-mediated cervicogenic headache. In view of the patient's advanced age and cardiovascular comorbidities, minimally invasive RFA was selected in preference to endoscopic or surgical interventions, with a view to minimising the risk of anaesthesia and tissue trauma.

On June 6, 2024, the patient received multi-segment radiofrequency ablation under ultrasound and fluoroscopic guidance. Targeted therapy was administered to the right C5 spinal nerve root, right C5/6 facet joint, left C7 nerve root, bilateral C2 spinal nerves (posterior branches), and bilateral C2/3 facet joints. Epidural steroid injections were administered at the right C5/6 facet joint, left C7 nerve root, and bilateral C2/3 facet joints simultaneously. Pulsed radiofrequency was administered to each target site using segment-specific parameters (voltage and duration) to mitigate nerve root compression and facet-mediated pain, thereby addressing the intricate pathology of cervical spondylotic radiculopathy.

**Figure 1:**
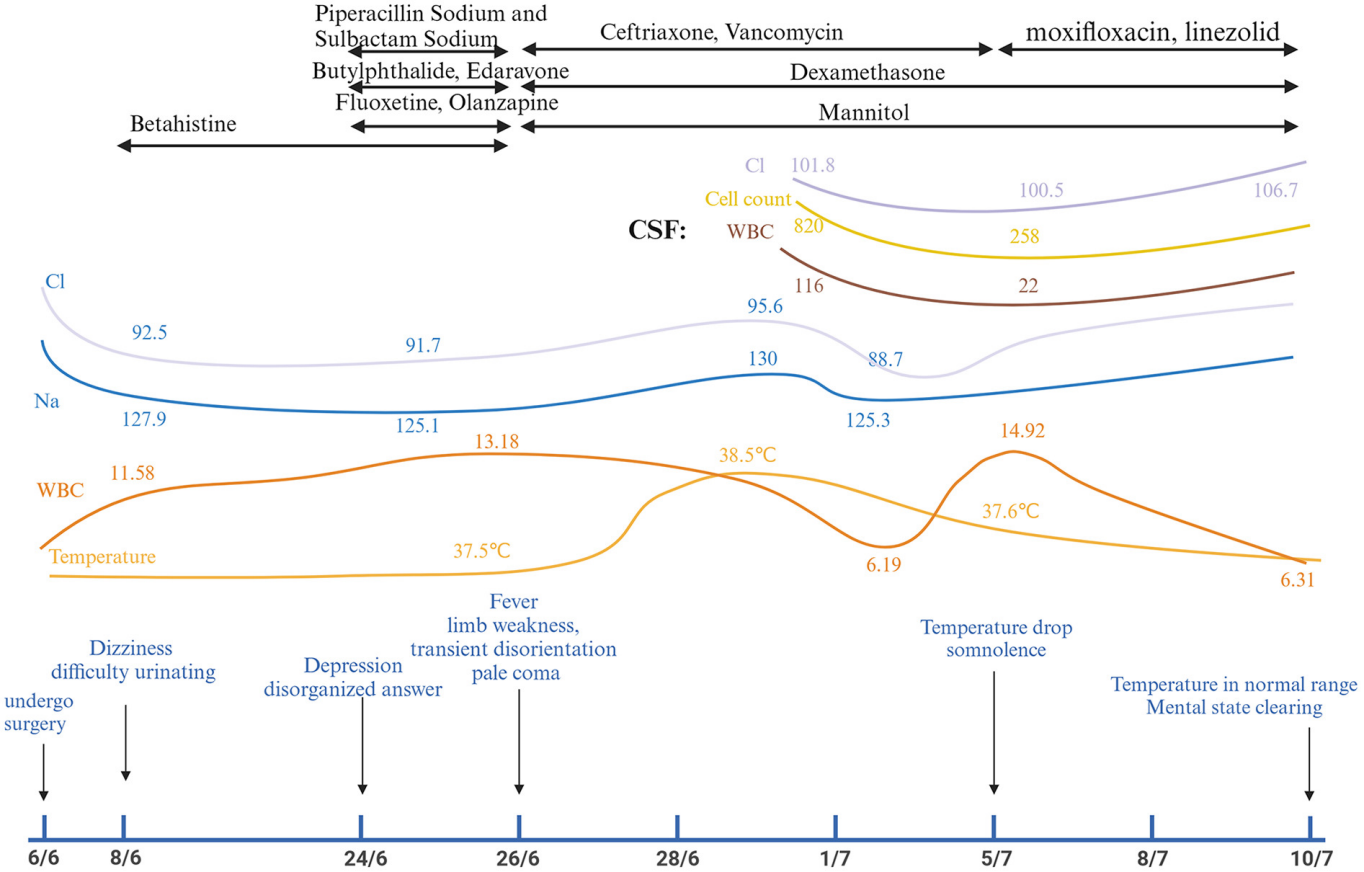
Timelines.

Postoperatively, the patient reported partial alleviation of cervical discomfort (VAS: 3/10). Two days post-procedure, she exhibited depressive symptoms, dizziness, and urinary retention, necessitating emergency evaluation. Her condition swiftly declined, presenting as fever, muscular weakness, altered mental status, and ultimately advancing to a comatose state marked by pallor and unresponsiveness. Figure 1 illustrates the sequence of symptom development.

## Investigations

**Figure 2:**
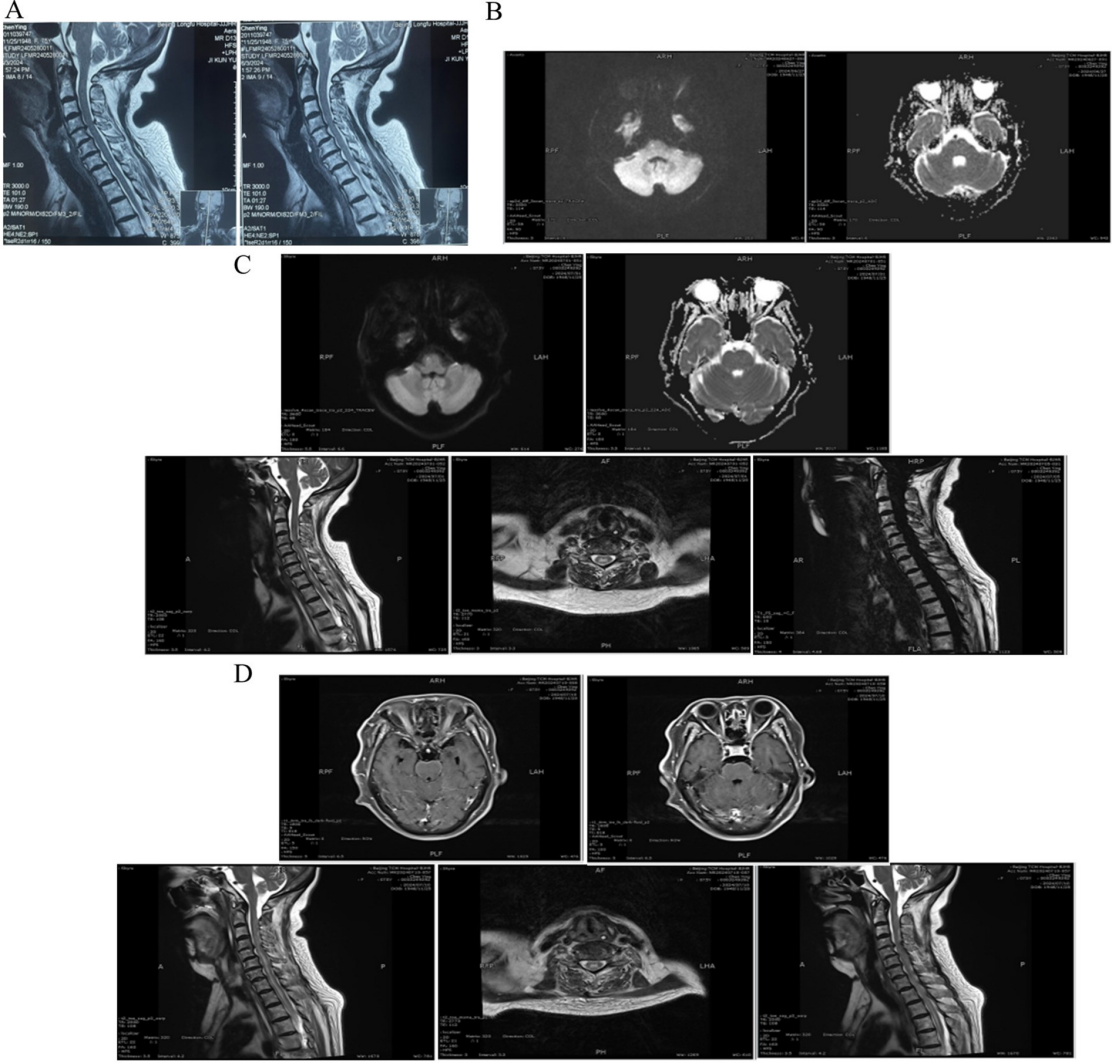
Cervical spine and brain MRI. **(A)** Preoperative cervical spine MRI: C3–7 spinal canal stenosis, C4–7 bilateral foraminal stenosis. **(B)** 27/6/2024 Brain MRI DWI and ADC image: the pons shows a minor, punctate, elevated signal. **(C)** 1/7/2024 Brain MRI DWI and ADC image: The cerebral bridge showed a small, punctate, elevated signal with no apparent diffusion coefficient (ADC) value change. 1/7/2024 Cervical spine MRI: The cervicothoracic medullary center at the C5–T2 level is distinguished by a striated high signal shadow on T2–weighted imaging. The dural sac at C4–C7 is compressed, the spinal canal is constricted at the horizontal level, the cervical medulla is compressed, and the paravertebral soft tissues show no abnormal signals. **(D)** 10/7/2024 Brain MRI T1WI: The meninges of the pontine and periaqueductal regions exhibit mild thickening and significant enhancement. 10/7/2024 Cervical spine MRI: No anomalous signals were detected in the cones; however, there is evidence of dural sac compression at C4–C7, spinal canal narrowing at that level, cervical medulla compression, and no abnormal signals in the paravertebral soft tissues. A prominent hyperintense shadow was noted on T2–weighted images throughout the cervical medulla and at the center of the thoracic medulla at the T1–5 level. The spinal cord was observed to be mildly thickened at the C4–T5 level.

At the time of emergency (8/6/2024), Laboratory tests found hyponatremia in addition to an elevated white blood cell count (WBC) and neutrophil percentage (NEUT%). Furthermore, the patient displayed grade 4 muscle strength in all four limbs, normal muscle tone, and no pathological signs. Twenty days later, the patient exhibited symptoms of fever and coma. The subject demonstrated positive responses to both the Babinski and Chaddock tests. Cerebrospinal fluid analysis revealed elevated WBC levels, higher protein concentration, lower glucose and chloride levels, all of which point to bacterial meningitis. MRI of the cervical spine revealed signal changes matching to myelitis. Among specific laboratory results were the following: 8/6/2024 White Blood Cells (WBC): 11.58 × 10^9^/L, Neutrophil Percentage (NEUT%): 93.8%, CRP: 0.25 mg/L, Sodium (Na^+^): 127.9 mmol/L, Chloride(Cl^−^): 92.5 mmol/L, Potassium (K^+^): 2.93 mmol/L. 2/7/2024 Cerebrospinal Fluid (CSF) Analysis: WBC Count:116 × 10^6^/L, Protein Concentration: >10,000 mg/L, Glucose Concentration:0.72 mmol/L, Chloride Concentration:101.8 mmol/L. 1/7/2024 Cervical spine MRI: Revealed C3–7 spinal stenosis, C4–7 bilateral neuroforaminal stenosis, and postoperatively, C5–T2 level intramedullary abnormal signals indicative of myelitis. Figure 1 and Supplementary 1 shows the chronology of laboratory experiments. Figure 2 shows imaging results in detail.

## Treatment

The patient received a regimen of antibiotics, hormone shock therapy, and dehydration management. The antibiotic regimen comprised piperacillin sodium, sulbactam sodium, ceftriaxone, vancomycin, moxifloxacin, and linezolid. Furthermore, hormone shock therapy and mannitol were utilized to reduce intracranial pressure. Figure 1 illustrates the treatment timeline.

## Outcome and follow–up

The patient's mental status enhanced, shifting from a pale coma to a state of somnolence. The patient started to react to verbal prompts and exhibited minor movements of the extremities away from the bed surface. The patient's mental status improved, shifting from a pale coma to a state of somnolence. The patient started to react to verbal prompts and exhibited minor movements of the extremities away from the bed surface. Furthermore, the patient's body temperature returned to normal. At the four-week follow-up, the neurological examination revealed significant recovery, with limb muscle strength improving to grade 4+/5, resolution of sensory deficits, and the absence of pathological reflexes (negative Babinski and Chaddock signs). Laboratory analyses indicated the normalization of serum electrolytes (Na^+^: 138 mmol/L; Cl^−^: 102 mmol/L) and inflammatory markers (WBC: 7.2 × 10^9^/L; CRP: 0.1 mg/L). The follow-up cervical MRI indicated a reduction in the previously noted intramedullary T2 hyperintensity from C5 to T2, stable spinal canal stenosis, and no signs of new lesions. The patient is still alive at the time of this report.

## Discussion

Two days after the operation, the patient first showed up to the hospital complaining of hyponatremia and trouble urinating. At this point, complications from cervical spine radiofrequency ablation were not initially considered. Acute cerebral infarction was ascribed from follow-up cranial MRI revealing lacunar infarcts in the brainstem and treated promptly. As the patient's condition deteriorated, manifesting as cognitive decline and persistent hyponatremia, a consultation with the Department of Psychosomatic Medicine was initiated to evaluate the possibility of a depressive state. Unfortunately, Fever started to cause suspicion about an intracranial infection, which finally resulted in an encephalomyelitis diagnosis. The Cerebrospinal fluid analysis of this patient suggested that infection must be present. On the causes of infection, firstly, the glucose–chloride level of Cerebrospinal fluid analysis considered tuberculosis to be highly probable, but no direct evidence was found on repeated laboratory investigations, we finally ruled out tuberculosis because the current treatment has been effective. Secondly, in consideration of the individual differences among patients, those with immunocompromised diseases such as diabetes mellitus exhibit a diminished capacity for resisting pathogens, rendering them more susceptible to infection. In the event that the patient has a pre–operative viral infection (e.g., coxsackie virus, herpes simplex virus, etc.), which may be induced by surgical stress and decreased immunity, this should also be taken into account. In any case, our treatments can address the majority of the issues raised in the appeal and are effective. Thirdly, from a monistic perspective, the tight temporal association between surgery and symptom onset (within 48 h), along with imaging findings of segmental spinal cord inflammation (C5–T2) near the ablation sites, strongly favors a postoperative complication. If the infection is considered to be caused by surgery, the most plausible infection source is intraoperative contamination due to inadequate aseptic technique, given that the procedure targeted extra-discal structures and imaging excluded discitis, dural tears, or direct spinal cord injury. This excludes the possibility of direct intraoperative needle injury to the spinal cord. However, postoperative imaging did not show signs of osteomyelitis, facet joint sepsis, or epidural abscess at the ablation sites, which makes the direct extension of infection from bone/joint structures to the spinal cord less likely, leaving the exact cause of spinal cord infection still uncertain.

Meanwhile, It is necessary to consider the possibility of etiologies not directly related to the surgery. For example, acute transverse myelitis (ATM), as a condition not directly tied to the procedure, should be included in the differential diagnosis. Although the timing of symptom onset is close to the surgery, ATM can sometimes be triggered by surgical stress or infection (6). Furthermore, while the likelihood of acute disseminated encephalomyelitis (ADEM) cannot be discounted. The rationale behind this observation is that ADEM generally manifests with the presence of multiple lesions in the brain's white matter. However, the brain MRI of the patient in question revealed only a minor punctate high signal in the pons, devoid of any discernible white matter lesions. This finding is not in alignment with the conventional imaging characteristics associated with ADEM (7). Finally, as previously discussed, there is a possibility that this patient had a primary infection that was not surgically related, and that this infection led to haematogenous dissemination. While the patient's comorbidities (hypertension, prior cerebral infarction, hyperlipidemia) could theoretically increase infection risk, preoperative screening ruled out pre-existing infectious foci, and the clinical course lacked systemic sepsis features, making *de novo* hematogenous spread unlikely. Moreover, this regional distribution of myelitis is inconsistent with hematogenous spread, which typically manifests as diffuse or multifocal lesions. The rapid postoperative symptom onset further supports a procedure-related etiology, which may be directly caused by surgical infection or triggered by surgery as a contributing factor. Nevertheless, the possibility of hematogenous spread of infection continued to be a cause for concern.

Notably, this case represents the first reported instance of cervical RFA–associated myelitis, highlighting a previously unrecognized complication (4). The exact mechanisms—likely involving needle tract microbial translocation or inflammatory mediator release—warrant further investigation to inform aseptic protocols and complication surveillance in minimally invasive spinal procedures. With a total duration of 24 days from symptom start to definitive diagnosis, the patient displayed a subtle beginning and a protracted disease course. This case emphasizes the possibility of major morbidity following cervical spine radiofrequency ablation. There are many postoperative cervical spine complications being reported, including long–term neurologic injury, cervical dislocation, incomplete quadriplegia, epidural abscess,subarachnoid hemorrhage,and dropped head syndrome (8–12), these are all something we should be aware of especially as emergency physicians.

## Conclusion

Though it is regarded as a minimally invasive surgery, cervical spine radiofrequency ablation carries some hazards including myelitis. It is imperative for emergency physicians to be aware of the presence of early symptoms of complications. This knowledge enables the prompt diagnosis and treatment of the patient. Furthermore, a more nuanced understanding of the patient's surgical history is crucial for the accurate identification of potential complications.

## Data Availability

The original contributions presented in the study are included in the article/Supplementary Material, further inquiries can be directed to the corresponding authors.
